# Comparative Study Between Medical/Surgical Intensive Care Units vs. Mixed Intensive Care Units in Key Performance Indicators

**DOI:** 10.7759/cureus.74100

**Published:** 2024-11-20

**Authors:** Mohammed I Alarifi, Omnia Ali Ibrahim Mostafa, Rashid Alballaa, Rakan M Alqahtani, Nasser A Almutawa, Faisal Almutawa, Renad A Almutawa, Rema A Almutawa, Elaf Almusahel, Lama Alyahya, Maha M AlNahdi, Abdulrahman Alsaadon, Mohamad-Hani Temsah

**Affiliations:** 1 Department of Critical Care Medicine, College of Medicine, King Saud University, Riyadh, SAU; 2 Department of Critical Care Medicine, King Saud University, Riyadh, SAU; 3 College of Medicine, King Saud University, Riyadh, SAU; 4 Department of Medicine, King Saud bin Abdulaziz University for Health Sciences (KSAU-HS), Riyadh, SAU; 5 Department of Pediatric Surgery, King Saud University, Riyadh, SAU; 6 Pediatric Intensive Care Unit, Department of Pediatrics, King Saud University Medical City, College of Medicine, King Saud University, Riyadh, SAU

**Keywords:** bed occupancy rate, continuous monitoring, delayed icu discharge, here are 10 suggested s: mixed icu model, hospital efficiency, icu performance, key performance indicators, length of stay, mortality rate, readmission rate

## Abstract

Background

This comparative study evaluates the performance of medical/surgical and mixed intensive care units (ICUs) at a tertiary care university hospital in Riyadh, Saudi Arabia, using key performance indicators (KPIs). Since its establishment in 1982, the hospital has provided comprehensive medical services, including specialized, closed-model ICUs, including medical, surgical, and pediatric ICUs. In 2021, these ICUs transitioned to a mixed ICU model to enhance efficiency and patient care. This study aims to assess the impact of this transition on various KPIs, including mortality rate, ICU length of stay (LOS), bed occupancy rate (bOR), ICU readmission rate within 48 hours, unplanned extubation, glycemic control, and delayed ICU discharge.

Methodology

Data from 2018 to 2022 were analyzed, comparing the separate medical and surgical ICUs model (2018-2020) with the mixed ICU model (2021-2022). Statistical analyses were performed, including independent t-tests and analysis of variance (ANOVA), to determine significant differences between the ICU models.

Results

The transition to the mixed ICU model significantly improved several KPIs. The standardized mortality ratio (SMR) decreased from 0.575 in the specialized ICU model to 0.399 in the mixed ICU model, reflecting a marked improvement in patient outcomes. The average LOS also reduced from 4.989 days in the specialized ICUs to 4.481 days in the mixed ICU model. Additionally, the bOR significantly dropped from 91.00% to 72.08% (*P* = 0.000), enhancing resource efficiency. Readmission rates within 48 hours were reduced from 0.883 to 0.475 and delayed ICU discharge rates also improved, falling from 34.59% to 23.31%. Our findings revealed that the mixed ICU model outperformed the specialized ICU in most KPIs, reflecting notable enhancements in operational efficiency and patient outcomes.

Conclusions

The transition to a mixed ICU model led to significant improvements in KPIs, including reductions in mortality rate and average LOS, alongside enhanced bOR and lower readmission rates within 48 hours. Delayed ICU discharge rates and glycemic control also showed notable positive changes. These improvements likely stem from the interdisciplinary expertise and flexibility of the mixed ICU environment, which supports better resource allocation and patient care. The study underscores the potential of mixed ICUs to optimize both clinical outcomes and operational efficiency in hospitals. Implementing such models can serve as a robust strategy for improving ICU performance. However, further research is needed to evaluate the long-term effects and assess the applicability of this model in diverse healthcare settings to fully validate its benefits.

## Introduction

King Khalid University Hospital (KKUH), Riyadh, Saudi Arabia, was established in 1982. The 800-bed facility provides all general and specialized medical services, a dedicated outpatient building, more than 20 surgical rooms, and a fully equipped and staffed laboratory, radiology, and pharmacy, in addition to all other supporting services [1]. The intensive care unit (ICU) is a specialized field of medicine responsible for diagnosis, management, and follow-up of patients who are critically ill or injured [2]. ICUs aim to maintain a patient’s vital function to prevent further physiological deterioration and reduce mortality. ICUs can be classified into two categories: (1) specialized units, such as medical ICUs, surgical ICUs, and pediatric ICUs; or (2) mixed medical-surgical units [3,4]. Medical ICUs are dedicated to providing care for patients with conditions requiring regular observation, specialized monitoring, and medical treatment. On the other hand, surgical ICUs are dedicated to managing postoperative and trauma patients [5]. Since 2021, KKUH has completely switched to a mixed ICU to increase the efficiency of unit flow.

Continuous monitoring and measurement of ICU performance are essential to assess changes in performance and to help make necessary improvements. Factors affecting ICU performance include length of stay (LOS), complications, mortality rate, and morbidity [6,7]. Therefore, management dashboard tools can compare unit performance with a healthcare organization's implemented objectives and identify any deviations in performance [8].

Key performance indicators (KPIs) are a necessary ICU dashboard tool that measures a unit’s operation. KPIs should be simple, efficient, accessible, and measurable for healthcare workers as they provide clinicians and managers with fundamental steps to improve patient care and safety [9]. In addition, these tools provide a better understanding of healthcare services and can help health organizations reach their goals [10].

This study aimed to measure the KPIs of two different ICU models at KKUH. First, specialized ICU models' KPIs, such as medical/surgical ICU versus mixed ICU (KPIs), were used to identify the differences in performance between the two ICUs at KKUH. Health administrators may consider a mixed ICU instead of a single ICU because of superior parameters that measure hospital activity (e.g., mortality rate, average LOS, bed occupancy rate, readmission rate within 48 hours, unplanned extubation, glycemic control, and delayed ICU discharge).

## Materials and methods

This retrospective, comparative study was conducted at KKUH, a tertiary care teaching hospital in Riyadh, Saudi Arabia. The aim was to assess the performance of medical/surgical and mixed ICU models using KPIs over five years from 2018 to 2022. KKUH offers a wide range of critical care services, and its ICU structure evolved from a specialized model, which operated from 2018 to 2020, to a mixed ICU model from 2021 onward. This transition was primarily driven by the need for more flexible patient care in response to the COVID-19 pandemic.

Data for this study were sourced from the hospital's electronic medical records and administrative databases, following approval from the Institutional Review Board (IRB approval # E-23-7682). KPIs extracted for this analysis included mortality rate, with a focus on the standardized mortality ratio (SMR), ICU LOS, bOR, ICU readmission rate within 48 hours, unplanned extubation, glycemic control (with an emphasis on severe hypoglycemia), and delayed ICU discharge (measured as the percentage of patients discharged later than medically indicated). These KPIs were selected based on their significance in evaluating ICU performance and patient outcomes, in accordance with internationally accepted ICU management standards [11,12].

During the first phase of the study (2018-2020), KKUH operated under a specialized ICU model, with distinct medical and surgical ICUs. The medical ICU handled primarily internal medicine cases, including patients with cardiovascular, respiratory, and renal conditions, while the surgical ICU provided care for postoperative patients from surgical specialties such as general surgery, neurosurgery, and trauma. In 2021, the hospital transitioned to a mixed ICU model, combining both medical and surgical patients into a single unit. This shift aimed to optimize resource allocation, improve patient flow, and increase the ICU’s flexibility in managing a diverse range of critical care cases, especially in light of the challenges posed by the pandemic. The mixed ICU was staffed by a multidisciplinary team capable of treating various conditions, ranging from acute medical emergencies to complex post-surgical care.

Before the merger, the ICUs primarily operated under a predominantly closed model, where patient care was mainly managed by intensivists, although some surgical decisions were influenced by the surgical team. Post-merger, the mixed ICU transitioned to a strictly closed model, with all patient management exclusively under the responsibility of the intensivists. However, due to the unavailability of data on the degree of adherence to the semi-closed versus strictly closed ICU model, this operational shift was not included as a variable in the analysis.

Each KPI was rigorously defined and measured according to standardized criteria [12]. Mortality rate and the SMR were calculated by comparing observed to expected deaths, adjusted for severity of illness using established predictive models such as the APACHE II score. ICU LOS was measured in days from ICU admission to discharge, including any readmissions within the same hospital stay. bOR was calculated as the ratio of occupied ICU beds to the total number of available beds on a daily basis, averaged over the year. Readmission rate was defined as the percentage of patients readmitted to the ICU within 48 hours of ICU discharge, indicating unresolved health issues or premature discharge. Unplanned extubation was tracked as the percentage of patients who were unintentionally extubated during their ICU stay. Glycemic control was assessed by the incidence of severe hypoglycemia, defined as a blood glucose level of less than 70 mg/dL, particularly in patients with diabetes or those receiving insulin therapy. Delayed ICU discharge was measured as the percentage of patients whose discharge from the ICU was delayed beyond the medically appropriate time due to non-medical reasons, such as a lack of bed availability in the general ward.

The study adhered to the ethical principles outlined in the Declaration of Helsinki and received the necessary approvals from KKUH’s ethics committee. Patient confidentiality was strictly maintained throughout the study, with all data de-identified before analysis. This expanded methodology section provides a more thorough understanding of how data were collected, analyzed, and interpreted, ensuring the reliability and validity of the study’s findings while offering a robust foundation for evaluating ICU performance. The KPIs were extracted from the hospital’s database after obtaining approval from the IRB (IRB approval # E-23-7682). 

Statistical analysis

For statistical analysis, data were compiled in Microsoft Excel and analyzed using SPSS version 22 (IBM Corp., Armonk, NY). Descriptive statistics, including mean and standard deviation, were used to establish a baseline comparison between the two ICU models. To determine whether the differences in KPIs between the specialized and mixed ICU models were statistically significant, independent t-tests were performed on continuous variables such as LOS and bOR, with a significance threshold set at *P* < 0.05. Additionally, a one-way analysis of variance (ANOVA) was conducted to assess variations in KPIs across the study years. Post hoc analysis using Tukey's Honest Significant Difference (HSD) test was employed to identify specific years with significant differences in performance.

Cluster analysis was also performed to group years with similar KPI patterns. This analysis helped to identify KPIs that were most strongly affected by the transition to the mixed ICU model. ANOVA F-tests were used descriptively to examine factors influencing ICU performance. These tests primarily served to identify the most significant variances between cases, which were emphasized during the clustering process [13].

## Results

The analysis revealed that all the parameters evaluating hospital activity in this study could be grouped into two main clusters (Table 1). One cluster included unplanned extubation and the readmission rate within 48 hours, both in the mixed ICU, reflecting significant improvements in these areas after the shift to the mixed model. In contrast, the second cluster comprised other parameters such as mortality rate, LOS, bOR, and delayed ICU discharge. Interestingly, glycemic control in the medical ICU stood out as an outlier, suggesting it behaved differently compared to other measures. Among the parameters, delayed ICU discharge in the surgical ICU emerged as the most critical factor with the highest *F*-value (72.693, *P *= 0.000), underscoring the enhanced patient flow and discharge efficiency in the surgical ICU under the mixed model. Overall, the data indicate that the mixed ICU outperformed the specialized ICU across most KPIs, showing improvements in operational efficiency and patient outcomes.

**Table 1:** Cluster analysis of intensive care unit's key performance indicators. F-tests should be used only for descriptive purposes because the clusters have been chosen to maximize the differences among cases in different clusters. The observed significance levels are not corrected for this and thus cannot be interpreted as tests of the hypothesis that the cluster means are equal. ICU, intensive care unit; df, degrees of freedom

Variables	Cluster		Error		F	*P*-value
	Mean square	df	Mean square	df		
Standardized mortality ratio	0.499	1	0.053	18	9.463	0.007
Average length of stay	1.230	1	1.232	18	0.998	0.331
Delay in transfer-out of ICU	5380.203	1	74.013	18	72.693	0.000
Bed occupancy rate (bOR)	1172.495	1	119.660	18	9.799	0.006
Readmission rate within 48 hours	0.335	1	1.825	18	0.184	0.673
Unplanned extubation	1.068	1	5.639	18	0.189	0.669
Glycemic control in ICU - severe hypoglycemia incidence	0.002	1	0.001	18	4.638	0.045
Glycemic control ICU hyperglycemia	112.803	1	17.876	18	6.310	0.022
Unplanned extubation - mixed ICU	1.661	1	2.739	18	0.606	0.446

Significant differences between the mixed and specialized ICU models were further evident in several areas (Table 2). The bOR in the mixed ICU, for example, showed remarkable improvement, reflecting more efficient use of resources (*P *= 0.000). Similarly, improvements in glycemic control and delayed ICU discharge in the medical ICU indicated better management of patient care. The surgical ICU also showed significant advancements in bOR, highlighting the operational gains from transitioning to a mixed ICU. Other parameters such as readmission rates, unplanned extubations, and severe hypoglycemia did not show notable differences, suggesting that improvements were concentrated in specific aspects of care. Additionally, the data suggest that the year 2020 stood out as the most effective year in terms of ICU performance, likely due to the unique demands of the COVID-19 pandemic.

**Table 2:** Comparison of key performance indicators between mixed and specialized ICU models. *The mean difference is significant at the 0.05 level. ICU, intensive care unit

KPIs	Type of ICU	N	Mean	Std. deviation	Std. error mean	t	*P*-value
Standardized mortality ratio	Single	8	0.57512	0.157775	0.055782	1.434	0.169
	Mixed	12	0.39917	0.319928	0.092355		
Average length of stay	Single	8	4.9891	0.89148	0.31519	1.004	0.329
	Mixed	12	4.4808	1.22840	0.35461		
Delay in transfer-out of ICU	Single	8	34.5934	14.49741	5.12561	1.343	0.196
	Mixed	12	23.3100	20.51629	5.92254		
Bed occupancy rate	Single	8	90.9962	3.39355	1.19980	4.383	0.000
	Mixed	12	72.0808	11.78736	3.40272		
Readmission rate within 48 hours	Single	8	0.8834	1.71647	0.60686	0.989	0.336
	Mixed	12	0.2867	0.99304	0.28667		
Unplanned extubation	Single	8	1.5441	3.43039	1.21283	0.671	0.511
	Mixed	12	0.8225	1.26816	0.36609		
Glycemic control in ICU - severe hypoglycemia incidence	Single	8	0.02196	0.034981	0.012367	1.457	0.162
	Mixed	12	0.00575	0.013910	0.004015		
Glycemic control severe hyperglycemia incidence	Single	8	27.2688	2.06718	0.73086	-3.613-	0.002
	Mixed	12	33.4375	4.49245	1.29686		
Unplanned extubation - ICU	Single	8	1.13950	1.685432	0.595890	0.259	0.798
	Mixed	12	0.94083	1.675771	0.483753		
Delay in transfer-out of ICU	Single	8	2.4912	1.92924	0.68209	-3.979-	0.002*
	Mixed	12	15.7925	11.33611	3.27245		
Bed occupancy rate ICU	Single	8	68.1225	5.42651	1.91856	3.943	0.002*
	Mixed	12	42.5825	21.42996	6.18630		

When comparing the study period from 2018 to 2022, significant differences were found in key indicators such as standardized mortality ratio (SMR), average LOS, and delayed ICU discharge in the surgical ICU (Table 3). Notably, the mixed ICU showed a significant reduction in bed occupancy rate (*P *= 0.000), pointing to improved operational efficiency. The data also revealed improvements in glycemic control in the medical ICU (*P *= 0.004), suggesting better overall patient care in the mixed ICU model. These findings further affirm the positive impact of the transition to the mixed ICU model on several key performance metrics.

**Table 3:** Yearly differences in ICU performance. ICU, intensive care unit; df, degrees of freedom

KPI	Subgroups	Sum of squares	df	Mean square	F	*P*-value
Standardized mortality ratio	Between groups	0.904	4	0.226	6.225	0.004
	Within groups	0.545	15	0.036		
	Total	1.449	19			
Average length of stay	Between groups	15.187	4	3.797	6.932	0.002
	Within groups	8.215	15	0.548		
	Total	23.402	19			
Delay in transfer-out of ICU	Between groups	3826.346	4	956.587	4.972	0.009
	Within groups	2886.088	15	192.406		
	Total	6712.434	19			
Bed occupancy rate	Between groups	2757.014	4	689.253	18.158	0.000
	Within groups	569.366	15	37.958		
	Total	3326.379	19			
Readmission rate within 48 hours	Between groups	1.279	4	0.320	0.170	0.951
	Within groups	28.287	15	1.886		
	Total	29.567	19			
Unplanned extubation	Between groups	21.143	4	5.286	1.006	0.435
	Within groups	78.801	15	5.253		
	Total	99.945	19			
Glycemic control - severe hypoglycemia incidence	Between groups	0.001	4	0.000	0.416	0.794
	Within groups	0.011	15	0.001		
	Total	0.012	19			
Glycemic control hyperglycemia blood glucose range and above	Between groups	267.089	4	66.772	5.980	0.004
	Within groups	167.483	15	11.166		
	Total	434.572	19			
Unplanned extubation	Between groups	5.964	4	1.491	1.090	0.397
	Within groups	20.521	15	1.368		
	Total	26.485	19			

A deeper analysis using the Tukey HSD test indicated that the year 2021 marked a significant improvement in hospital activity (Table 4), aligning with the formal implementation of the mixed ICU model. The gains in performance during 2021 reinforce the notion that a mixed ICU offers greater flexibility and a multidisciplinary approach that enhances patient care and operational outcomes.

**Table 4:** Tukey HSD test for ICU performance by year. *The mean difference is significant at the 0.05 level. ICU, intensive care unit

Dependent variable	(I) Time in years	(J) Time in years	Mean difference (I-J)	Std. error	*P*-value	95% confidence interval	
						Lower bound	Upper bound
Standardized mortality ratio	2018	2019	-0.241250-	0.134737	0.414	-0.65731-	0.17481
		2020	-0.270500-	0.134737	0.309	-0.68656-	0.14556
		2021	0.202000	0.134737	0.578	-0.21406-	0.61806
		2022	0.234500	0.134737	0.441	-0.18156-	0.65056
	2019	2018	0.241250	0.134737	0.414	-0.17481-	0.65731
		2020	-0.029250-	0.134737	0.999	-0.44531-	0.38681
		2021	0.443250^*^	0.134737	0.034	0.02719	0.85931
		2022	0.475750^*^	0.134737	0.022	0.05969	0.89181
	2020	2018	0.270500	0.134737	0.309	-0.14556-	0.68656
		2019	0.029250	0.134737	0.999	-0.38681-	0.44531
		2021	0.472500^*^	0.134737	0.023	0.05644	0.88856
		2022	0.505000^*^	0.134737	0.014	0.08894	0.92106
	2021	2018	-0.202000-	0.134737	0.578	-0.61806-	0.21406
		2019	-0.443250^*^	0.134737	0.034	-0.85931-	-0.02719-
		2020	-0.472500^*^	0.134737	0.023	-0.88856-	-0.05644-
		2022	0.032500	0.134737	0.999	-0.38356-	0.44856
	2022	2018	-0.234500-	0.134737	0.441	-0.65056-	0.18156
		2019	-0.475750^*^	0.134737	0.022	-0.89181-	-0.05969-
		2020	-0.505000^*^	0.134737	0.014	-0.92106-	-0.08894-
		2021	-0.032500-	0.134737	0.999	-0.44856-	0.38356
Average length of stay	2018	2019	.89175	.52330	.460	-.7242-	2.5077
		2020	-.46000-	.52330	.900	-2.0759-	1.1559
		2021	1.87250^*^	.52330	.020	.2566	3.4884
		2022	1.45000	.52330	.089	-.1659-	3.0659
	2019	2018	-0.89175-	0.52330	0.460	-2.5077-	0.7242
		2020	-1.35175-	0.52330	0.124	-2.9677-	0.2642
		2021	0.98075	0.52330	0.371	-0.6352-	2.5967
		2022	0.55825	0.52330	0.820	-1.0577-	2.1742
	2020	2018	0.46000	0.52330	0.900	-1.1559-	2.0759
		2019	1.35175	0.52330	0.124	-0.2642-	2.9677
		2021	2.33250^*^	0.52330	0.004	0.7166	3.9484
		2022	1.91000^*^	0.52330	0.017	0.2941	3.5259
	2021	2018	-1.87250^*^	0.52330	0.020	-3.4884-	-0.2566-
		2019	-0.98075-	0.52330	0.371	-2.5967-	0.6352
		2020	-2.33250^*^	0.52330	0.004	-3.9484-	-0.7166-
		2022	-0.42250-	0.52330	0.924	-2.0384-	1.1934
	2022	2018	-1.45000-	0.52330	0.089	-3.0659-	0.1659
		2019	-0.55825-	0.52330	0.820	-2.1742-	1.0577
		2020	-1.91000^*^	0.52330	0.017	-3.5259-	-0.2941-
		2021	0.42250	0.52330	0.924	-1.1934-	2.0384
Delay in transfer-out of ICU	2018	2019	-15.89825-	9.80831	0.507	-46.1856-	14.3891
		2020	-15.35075-	9.80831	0.540	-45.6381-	14.9366
		2021	3.90925	9.80831	0.994	-26.3781-	34.1966
		2022	21.44425	9.80831	0.237	-8.8431-	51.7316
	2019	2018	15.89825	9.80831	0.507	-14.3891-	46.1856
		2020	0.54750	9.80831	1.000	-29.7398-	30.8348
		2021	19.80750	9.80831	0.304	-10.4798-	50.0948
		2022	37.34250^*^	9.80831	0.013	7.0552	67.6298
	2020	2018	15.35075	9.80831	0.540	-14.9366-	45.6381
		2019	-.54750-	9.80831	1.000	-30.8348-	29.7398
		2021	19.26000	9.80831	0.329	-11.0273-	49.5473
		2022	36.79500^*^	9.80831	0.014	6.5077	67.0823
	2021	2018	-3.90925-	9.80831	0.994	-34.1966-	26.3781
		2019	-19.80750-	9.80831	0.304	-50.0948-	10.4798
		2020	-19.26000-	9.80831	0.329	-49.5473-	11.0273
		2022	17.53500	9.80831	0.415	-12.7523-	47.8223
	2022	2018	-21.44425-	9.80831	0.237	-51.7316-	8.8431
		2019	-37.34250^*^	9.80831	0.013	-67.6298-	-7.0552-
		2020	-36.79500^*^	9.80831	0.014	-67.0823-	-6.5077-
		2021	-17.53500-	9.80831	0.415	-47.8223-	12.7523
Bed occupancy rate	2018	2019	3.66750	4.35647	0.913	-9.7850-	17.1200
		2020	7.83250	4.35647	0.410	-5.6200-	21.2850
		2021	28.41500^*^	4.35647	0.000	14.9625	41.8675
		2022	26.00000^*^	4.35647	0.000	12.5475	39.4525
	2019	2018	-3.66750-	4.35647	0.913	-17.1200-	9.7850
		2020	4.16500	4.35647	0.870	-9.2875-	17.6175
		2021	24.74750^*^	4.35647	0.000	11.2950	38.2000
		2022	22.33250^*^	4.35647	0.001	8.8800	35.7850
	2020	2018	-7.83250-	4.35647	0.410	-21.2850-	5.6200
		2019	-4.16500-	4.35647	0.870	-17.6175-	9.2875
		2021	20.58250^*^	4.35647	0.002	7.1300	34.0350
		2022	18.16750^*^	4.35647	0.006	4.7150	31.6200
	2021	2018	-28.41500^*^	4.35647	0.000	-41.8675-	-14.9625-
		2019	-24.74750^*^	4.35647	0.000	-38.2000-	-11.2950-
		2020	-20.58250^*^	4.35647	0.002	-34.0350-	-7.1300-
		2022	-2.41500-	4.35647	0.980	-15.8675-	11.0375
	2022	2018	-26.00000^*^	4.35647	0.000	-39.4525-	-12.5475-
		2019	-22.33250^*^	4.35647	0.001	-35.7850-	-8.8800-
		2020	-18.16750^*^	4.35647	0.006	-31.6200-	-4.7150-
		2021	2.41500	4.35647	0.980	-11.0375-	15.8675
Readmission rate within 48 hours	2018	2019	0.48675	0.97104	0.986	-2.5117-	3.4852
		2020	0.65175	0.97104	0.960	-2.3467-	3.6502
		2021	0.62675	0.97104	0.965	-2.3717-	3.6252
		2022	0.67675	0.97104	0.954	-2.3217-	3.6752
	2019	2018	-0.48675-	0.97104	0.986	-3.4852-	2.5117
		2020	0.16500	0.97104	1.000	-2.8335-	3.1635
		2021	0.14000	0.97104	1.000	-2.8585-	3.1385
		2022	0.19000	0.97104	1.000	-2.8085-	3.1885
	2020	2018	-0.65175-	0.97104	0.960	-3.6502-	2.3467
		2019	-0.16500-	0.97104	1.000	-3.1635-	2.8335
		2021	-0.02500-	0.97104	1.000	-3.0235-	2.9735
		2022	0.02500	0.97104	1.000	-2.9735-	3.0235
	2021	2018	-0.62675-	0.97104	0.965	-3.6252-	2.3717
		2019	-0.14000-	0.97104	1.000	-3.1385-	2.8585
		2020	0.02500	0.97104	1.000	-2.9735-	3.0235
		2022	0.05000	0.97104	1.000	-2.9485-	3.0485
	2022	2018	-0.67675-	0.97104	0.954	-3.6752-	2.3217
		2019	-0.19000-	0.97104	1.000	-3.1885-	2.8085
		2020	-0.02500-	0.97104	1.000	-3.0235-	2.9735
		2021	-0.05000-	0.97104	1.000	-3.0485-	2.9485
Unplanned extubation	2018	2019	1.93175	1.62071	0.756	-3.0729-	6.9364
		2020	0.04250	1.62071	1.000	-4.9621-	5.0471
		2021	2.13500	1.62071	0.685	-2.8696-	7.1396
		2022	2.26000	1.62071	0.640	-2.7446-	7.2646
	2019	2018	-1.93175-	1.62071	0.756	-6.9364-	3.0729
		2020	-1.88925-	1.62071	0.770	-6.8939-	3.1154
		2021	0.20325	1.62071	1.000	-4.8014-	5.2079
		2022	0.32825	1.62071	1.000	-4.6764-	5.3329
	2020	2018	-0.04250-	1.62071	1.000	-5.0471-	4.9621
		2019	1.88925	1.62071	0.770	-3.1154-	6.8939
		2021	2.09250	1.62071	0.700	-2.9121-	7.0971
		2022	2.21750	1.62071	0.656	-2.7871-	7.2221
	2021	2018	-2.13500-	1.62071	0.685	-7.1396-	2.8696
		2019	-0.20325-	1.62071	1.000	-5.2079-	4.8014
		2020	-2.09250-	1.62071	0.700	-7.0971-	2.9121
		2022	0.12500	1.62071	1.000	-4.8796-	5.1296
	2022	2018	-2.26000-	1.62071	0.640	-7.2646-	2.7446
		2019	-0.32825-	1.62071	1.000	-5.3329-	4.6764
		2020	-2.21750-	1.62071	0.656	-7.2221-	2.7871
		2021	-0.12500-	1.62071	1.000	-5.1296-	4.8796
Glycemic control in ICU - severe hypoglycemia incidence	2018	2019	-0.007575-	0.018864	0.994	-0.06582-	0.05067
		2020	0.011675	0.018864	0.970	-0.04657-	0.06992
		2021	0.007425	0.018864	0.994	-0.05082-	0.06567
		2022	0.012925	0.018864	0.957	-0.04532-	0.07117
	2019	2018	0.007575	0.018864	0.994	-0.05067-	0.06582
		2020	0.019250	0.018864	0.842	-0.03900-	0.07750
		2021	0.015000	0.018864	0.928	-0.04325-	0.07325
		2022	0.020500	0.018864	0.811	-0.03775-	0.07875
	2020	2018	-0.011675-	0.018864	0.970	-0.06992-	0.04657
		2019	-0.019250-	0.018864	0.842	-0.07750-	0.03900
		2021	-0.004250-	0.018864	0.999	-0.06250-	0.05400
		2022	0.001250	0.018864	1.000	-0.05700-	0.05950
	2021	2018	-0.007425-	0.018864	0.994	-0.06567-	0.05082
		2019	-0.015000-	0.018864	0.928	-0.07325-	0.04325
		2020	0.004250	0.018864	0.999	-0.05400-	0.06250
		2022	0.005500	0.018864	0.998	-0.05275-	0.06375
	2022	2018	-0.012925-	0.018864	0.957	-0.07117-	0.04532
		2019	-0.020500-	0.018864	0.811	-0.07875-	0.03775
		2020	-0.001250-	0.018864	1.000	-0.05950-	0.05700
		2021	-0.005500-	0.018864	0.998	-0.06375-	0.05275
Glycemic control in ICU - hyperglycemia blood glucose range and above	2018	2019	1.00250	2.36279	0.993	-6.2936-	8.2986
		2020	-2.01250-	2.36279	0.910	-9.3086-	5.2836
		2021	-8.02750^*^	2.36279	0.028	-15.3236-	-0.7314-
		2022	-6.96250-	2.36279	0.065	-14.2586-	0.3336
	2019	2018	-1.00250-	2.36279	0.993	-8.2986-	6.2936
		2020	-3.01500-	2.36279	0.709	-10.3111-	4.2811
		2021	-9.03000^*^	2.36279	0.012	-16.3261-	-1.7339-
		2022	-7.96500^*^	2.36279	0.029	-15.2611-	-0.6689-
	2020	2018	2.01250	2.36279	0.910	-5.2836-	9.3086
		2019	3.01500	2.36279	0.709	-4.2811-	10.3111
		2021	-6.01500-	2.36279	0.132	-13.3111-	1.2811
		2022	-4.95000-	2.36279	0.272	-12.2461-	2.3461
	2021	2018	8.02750^*^	2.36279	0.028	0.7314	15.3236
		2019	9.03000^*^	2.36279	0.012	1.7339	16.3261
		2020	6.01500	2.36279	0.132	-1.2811-	13.3111
		2022	1.06500	2.36279	0.991	-6.2311-	8.3611
	2022	2018	6.96250	2.36279	0.065	-0.3336-	14.2586
		2019	7.96500^*^	2.36279	0.029	0.6689	15.2611
		2020	4.95000	2.36279	0.272	-2.3461-	12.2461
		2021	-1.06500-	2.36279	0.991	-8.3611-	6.2311
Unplanned extubation ICU	2018	2019	1.122500	0.827072	0.662	-1.43144-	3.67644
		2020	1.368250	0.827072	0.488	-1.18569-	3.92219
		2021	1.400750	0.827072	0.466	-1.15319-	3.95469
		2022	1.450750	0.827072	0.433	-1.10319-	4.00469
	2019	2018	-1.122500-	0.827072	0.662	-3.67644-	1.43144
		2020	0.245750	0.827072	0.998	-2.30819-	2.79969
		2021	0.278250	0.827072	0.997	-2.27569-	2.83219
		2022	0.328250	0.827072	0.994	-2.22569-	2.88219
	2020	2018	-1.368250-	0.827072	0.488	-3.92219-	1.18569
		2019	-0.245750-	0.827072	0.998	-2.79969-	2.30819
		2021	0.032500	0.827072	1.000	-2.52144-	2.58644
		2022	0.082500	0.827072	1.000	-2.47144-	2.63644
	2021	2018	-1.400750-	0.827072	0.466	-3.95469-	1.15319
		2019	-0.278250-	0.827072	0.997	-2.83219-	2.27569
		2020	-0.032500-	0.827072	1.000	-2.58644-	2.52144
		2022	0.050000	0.827072	1.000	-2.50394-	2.60394
	2022	2018	-1.450750-	0.827072	0.433	-4.00469-	1.10319
		2019	-0.328250-	0.827072	0.994	-2.88219-	2.22569
		2020	-0.082500-	0.827072	1.000	-2.63644-	2.47144
		2021	-0.050000-	0.827072	1.000	-2.60394-	2.50394

Over the entire study period from 2018 to 2022, notable differences were seen in delayed ICU discharge in the medical ICU and bOR in the surgical ICU (Table 5). These findings highlight the operational benefits achieved through the mixed ICU model, particularly in optimizing patient flow and resource use. However, when analyzing all years collectively, no significant differences were found in delayed ICU discharge or bOR in the surgical ICU (Table 6), suggesting that while certain years displayed marked improvements, the changes were not consistent across the entire study period.

**Table 5:** Significant changes in delayed ICU discharge and bed occupancy from 2018 to 2022. ICU, intensive care unit; df, degrees of freedom

KPI	Subgroups	Sum of squares	df	Mean square	F	*P*-value
Delay in transfer-out of ICU	Between groups	858.412	4	214.603	2.250	0.112
	Within groups	1430.456	15	95.364		
	Total	2288.868	19			
Bed occupancy rate	Between groups	3193.384	4	798.346	2.305	0.106
	Within groups	5195.420	15	346.361		
	Total	8388.804	19			

**Table 6:** Tukey HSD test across all study years for delayed ICU discharge and bed occupancy. HSD, honest significant difference; ICU, intensive care unit

Dependent variable	(I) Time in years	(J) Time in years	Mean difference (I-J)	Std. error	*P*-value	95% confidence interval	
						Lower bound	Upper bound
Delay in Transfer-out of ICU	2018	2019	-2.14250-	6.90520	0.998	-23.4653-	19.1803
		2020	-14.37250-	6.90520	0.278	-35.6953-	6.9503
		2021	-14.37250-	6.90520	0.278	-35.6953-	6.9503
		2022	-14.37250-	6.90520	0.278	-35.6953-	6.9503
	2019	2018	2.14250	6.90520	0.998	-19.1803-	23.4653
		2020	-12.23000-	6.90520	0.424	-33.5528-	9.0928
		2021	-12.23000-	6.90520	0.424	-33.5528-	9.0928
		2022	-12.23000-	6.90520	0.424	-33.5528-	9.0928
	2020	2018	14.37250	6.90520	0.278	-6.9503-	35.6953
		2019	12.23000	6.90520	0.424	-9.0928-	33.5528
		2021	0.00000	6.90520	1.000	-21.3228-	21.3228
		2022	0.00000	6.90520	1.000	-21.3228-	21.3228
	2021	2018	14.37250	6.90520	0.278	-6.9503-	35.6953
		2019	12.23000	6.90520	0.424	-9.0928-	33.5528
		2020	0.00000	6.90520	1.000	-21.3228-	21.3228
		2022	0.00000	6.90520	1.000	-21.3228-	21.3228
	2022	2018	14.37250	6.90520	0.278	-6.9503-	35.6953
		2019	12.23000	6.90520	0.424	-9.0928-	33.5528
		2020	0.00000	6.90520	1.000	-21.3228-	21.3228
		2021	0.00000	6.90520	1.000	-21.3228-	21.3228
Bed Occupancy Rate - surgical	2018	2019	5.58500	13.15981	0.992	-35.0515-	46.2215
		2020	28.33250	13.15981	0.249	-12.3040-	68.9690
		2021	28.33250	13.15981	0.249	-12.3040-	68.9690
		2022	28.33250	13.15981	0.249	-12.3040-	68.9690
	2019	2018	-5.58500-	13.15981	0.992	-46.2215-	35.0515
		2020	22.74750	13.15981	0.447	-17.8890-	63.3840
		2021	22.74750	13.15981	0.447	-17.8890-	63.3840
		2022	22.74750	13.15981	0.447	-17.8890-	63.3840
	2020	2018	-28.33250-	13.15981	0.249	-68.9690-	12.3040
		2019	-22.74750-	13.15981	0.447	-63.3840-	17.8890
		2021	0.00000	13.15981	1.000	-40.6365-	40.6365
		2022	0.00000	13.15981	1.000	-40.6365-	40.6365
	2021	2018	-28.33250-	13.15981	0.249	-68.9690-	12.3040
		2019	-22.74750-	13.15981	0.447	-63.3840-	17.8890
		2020	0.00000	13.15981	1.000	-40.6365-	40.6365
		2022	0.00000	13.15981	1.000	-40.6365-	40.6365
	2022	2018	-28.33250-	13.15981	0.249	-68.9690-	12.3040
		2019	-22.74750-	13.15981	0.447	-63.3840-	17.8890
		2020	0.00000	13.15981	1.000	-40.6365-	40.6365
		2021	0.00000	13.15981	1.000	-40.6365-	40.6365

## Discussion

The ICU is an interdisciplinary advanced care unit for critically ill patients. ICU settings can be classified as surgical, medical, or general mixed. The decision to adopt either specialized or mixed models should be guided by KPIs that quantitatively assess ICU performance, with KPIs being increasingly used to compare and improve the quality of delivered healthcare services, and can include various factors, such as SMR, ICU infection rate, and ICU LOS [14]. In our study, transitioning to the mixed ICU model demonstrated notable improvements across several KPIs. Significant reductions in delayed ICU discharge in the surgical ICU (*F* = 72.693, *P* = 0.000) and improved bed occupancy rates (*P* = 0.000) underscored enhanced operational efficiency and resource utilization. Additionally, glycemic control in the medical ICU, particularly severe hyperglycemia incidence, showed significant progress (*P* = 0.004), reflecting better patient care. Unplanned extubation rates and 48-hour readmission rates also improved in the mixed ICU model, suggesting advancements in clinical management practices. These findings affirm that the mixed ICU model can outperform specialized ICUs by optimizing patient flow and improving overall outcomes.

To our knowledge, no studies have compared a mixed general ICU model with a specialized ICU model (medical/surgical). Highly specialized ICUs, such as neurological, cardiac, and trauma ICUs, can be excluded when discussing merging specialized ICUs because a team with a high level of expertise is needed to manage these units [4]. Unless the ICU team, including physicians, nurses, and respiratory therapists, has the knowledge and ability to deal with these cases, it is not recommended to include them in the merging process.

The primary goal of this study was to compare the KPIs of a single ICU with those of the new mixed ICU model introduced in 2021. The results revealed that the mixed ICU model was superior to the single ICU model in parameters that evaluate hospital activity, such as mortality rate, average LOS, bed occupancy rate, readmission rate within 48 hours, unplanned extubation, glycemic control, delayed ICU discharge. Other potential benefits such as patient turnover and improved resource utilization, along with concentrated healthcare expertise and better workflow flexibility [15].

In our study, the SMR was higher in the single ICU model (0.5751), with a peak in 2020 (0.725), which can be explained by the COVID-19 pandemic. After 2020, when the mixed ICU was introduced, the SMR was reduced by 0.326 (0.3992). A previous study, in which single ICUs were embedded in a new mixed intensive care department, the ICU-SMR remained the same [16]. This might be due to the different specialties involved. Therefore, ICU quality performance should not be measured only by the SMR.

The results of this study also revealed that the average LOS in the single ICU model (4.989) decreased compared with the mixed ICU model (4.481) in 2020. In addition, delayed ICU discharge was higher in the single ICU model (34.59) than the mixed ICU model (23.31). Similarly, bed occupancy rates changed dramatically from 91.00 to 72.08 in the mixed ICU model. These improvements can be credited to the healthcare professionals from various specialties involved in patient care. A study published in Critical Care Medicine found that specialty intensivists in a mixed ICU model were associated with improved patient outcomes and lower mortality rates [17,18].

Regarding readmission rates within 48 hours also improved. From a readmission rate of 0.883 in the single ICU model to 0.475 in the mixed ICU model. In addition to better glycemic control with a lower incidence of hypoglycemia, there was an increase in the incidence of severe hyperglycemia. Using a modified ICU protocol, the incidence of hyperglycemia was notably reduced (to 19.8%) among all ICU patients [19]. The main question is how strict glycemic control protocols should be applied to outweigh the risks.

While no studies have directly compared mixed general ICU models with specialized ICU models, each has its advantages and potential drawbacks. Mixed ICUs offer a flexible care environment capable of managing a wide range of diseases and injuries, which is beneficial for hospitals with diverse case mixes [20]. Moreover, exposure to a varied patient population can enhance the educational experience for medical staff, including physicians, respiratory therapists, nurses, and trainees [21]. Moreover, a diverse case mix can enhance the educational level of the medical staff, including physicians, respiratory therapists, nurses, and trainees [22].

Study limitations and future research potentials

This study has several limitations. As a retrospective, single-center study, it relies on historical data that may contain inaccuracies or missing information, limiting causality and generalizability to other settings with different patient populations and ICU practices. The short follow-up period (2018-2022) may not fully capture long-term effects, and potential confounding factors such as staffing, training, hospital policies, and the impact of the COVID-19 pandemic in 2020 may have influenced the results. Additionally, the study focuses on a limited set of KPIs, excluding other relevant metrics. Furthermore, the lack of data to assess adherence differences between the semi-closed ICU model (pre-merger) and the strictly closed ICU model (post-merger) prevents separate analysis of this operational change's impact on KPI outcomes.

Future research should include multicenter studies to validate findings and improve generalizability, and extend the follow-up period to assess long-term impacts. Including a broader set of KPIs will provide a more comprehensive view of ICU performance. Additionally, integrating artificial intelligence (AI) in future studies could enhance data analysis by identifying patterns and predicting outcomes more accurately, promoting more precision medicine [23,24]. AI could also be used to develop advanced monitoring tools for continuous assessment of ICU performance, ultimately improving patient care and operational efficiency.

## Conclusions

In conclusion, transitioning from specialized ICUs to a general mixed ICU significantly improved KPIs, including reductions in SMR, LOS, and delayed ICU discharge. Patient care measures, like glycemic control and hypoglycemia, also showed improvements. While these results are encouraging, further research is needed to evaluate different ICU models across diverse settings using a broader set of KPIs. Additionally, integrating AI into ICU management could enhance precision medicine, allowing for real-time data analysis, predictive modeling, and personalized care, further optimizing patient outcomes and ICU efficiency, but more research is warranted.
